# Risk factors of brain metastases in completely resected pathological stage IIIA-N2 non-small cell lung cancer

**DOI:** 10.1186/1748-717X-7-119

**Published:** 2012-07-30

**Authors:** Xiao Ding, Honghai Dai, Zhouguang Hui, Wei Ji, Jun Liang, Jima Lv, Zongmei Zhou, Weibo Yin, Jie He, Luhua Wang

**Affiliations:** 1 Department of Radiation Oncology, Cancer Hospital & Institute, Chinese Academy of Medical Sciences and Peking Union Medical College, Pan jia yuan nan li 17#, Chao yang District, Beijing, 10021, China; 2 Department of Thoracic Surgery, Cancer Hospital & Institute, Chinese Academy of Medical Sciences and Peking Union Medical College, Beijing, China; 3 Department of oncology, Provincial Hospital Affiliated to Shandong University, Jinan, China

**Keywords:** Non-small cell lung cancer, Brain metastases, Prophylactic cranial irradiation, Risk factors, Non-squamous cell cancer, Lymph node ratio

## Abstract

**Background:**

Brain metastases (BM) is one of the most common failures of locally advanced non-small cell lung cancer (LA-NSCLC) after combined-modality therapy. The outcome of trials on prophylactic cranial irradiation (PCI) has prompted us to identify the highest-risk subset most likely to benefit from PCI. Focusing on patients with completely resected pathological stage IIIA-N2 (pIIIA-N2) NSCLC, we aimed to assess risk factors of BM and to define the highest-risk subset.

**Methods:**

Between 2003 and 2005, the records of 217 consecutive patients with pIIIA-N2 NSCLC in our institution were reviewed. The cumulative incidence of BM was estimated using the Kaplan–Meier method, and differences between the groups were analyzed using log-rank test. Multivariate Cox regression analysis was applied to assess risk factors of BM.

**Results:**

Fifty-three (24.4 %) patients developed BM at some point during their clinical course. On multivariate analysis, non-squamous cell cancer (relative risk [RR]: 4.13, 95 % CI: 1.86–9.19; P = 0.001) and the ratio of metastatic to examined nodes or lymph node ratio (LNR) ≥ 30 % (RR: 3.33, 95 % CI: 1.79–6.18; P = 0.000) were found to be associated with an increased risk of BM. In patients with non-squamous cell cancer and LNR ≥ 30 %, the 5-year actuarial risk of BM was 57.3 %.

**Conclusions:**

In NSCLC, patients with completely resected pIIIA-N2 non-squamous cell cancer and LNR ≥ 30 % are at the highest risk for BM, and are most likely to benefit from PCI. Further studies are warranted to investigate the effect of PCI on this subset of patients.

## Background

Non-small cell lung cancer (NSCLC) comprises approximately 85 % of lung cancer. Locally advanced (LA)-NSCLC comprises approximately 31–44 % of NSCLC. The risk of developing brain metastases (BM) in patients with early stage NSCLC is 10 %.[1] However, the risk of BM after treatment for LA-NSCLC is much higher, approximately 30–50 %.[1-6] BM is a devastating issue with a striking impact on survival and quality of life.

Advances in surgical and radiation techniques have diminished locoregional relapse of LA-NSCLC. Systemic chemotherapy has reduced the risk of extracranial metastases. Combined-modality therapy significantly increases survival. Recent studies employing multimodality therapy have reported median survival ranging from 20 to 43 months and 3-year survival rates of 34–63 % for LA-NSCLC.[7-13] However, chemotherapy has limited impact on BM because drugs do not easily penetrate the blood–brain barrier (BBB), which leaves the brain relatively undertreated.[5,14,15] The risk of BM increases as survival improves. Several studies have demonstrated that longer survival for patients with LA-NSCLC is associated with an increased incidence of BM, and that BM becomes a rising concern, detrimental to survival.[15,16] Therefore, decreasing the risk of BM becomes increasingly significant for achieving prolonged survival.

Prophylactic cranial irradiation (PCI) has shown effectiveness in small-cell lung cancer (SCLC). Notwithstanding a decrease in the incidence of BM, randomized trials have failed to prove the survival benefit from PCI in NSCLC.[17-20] Death from local and extracranial progression may have overwhelmed any apparent benefit from PCI. The outcome of RTOG 0214 in the modern era of combined-modality therapy, which implies that not all patients with LA-NSCLC should receive PCI, has prompted us to identify the subset, at the highest risk of BM, and most likely to benefit from PCI. Such candidates are most likely to be found in stage IIIA (N2), in that early stage has a relatively low BM risk, while stage IIIB has a relatively poor locoregional and extracranial control to obscure any potential survival benefits that PCI may have conferred.

Previous studies on risk factors of BM in NSCLC often have conflicting results and heterogeneous populations. We focused on patients with completely resected pathological stage IIIA-N2 (pIIIA-N2) NSCLC, and assessed risk factors for developing BM. Finally, we defined the highest-risk subset most likely to benefit from PCI.

## Methods

### Patients

We reviewed the records of 221 consecutive patients with completely resected pIIIA-N2 NSCLC who had survived no less than 4 months after surgery between January 2003 and December 2005 in our institution. Stage was recorded based on the American Joint Committee on Cancer staging system (6^th^).[21] Patients who presented with synchronous primary tumors, or had a prior history of lung cancer were excluded from this study. Medical records and follow-up data were reviewed to obtain patient and treatment characteristics, and to score recurrence pattern after surgery. All patients had negative brain computed tomography (CT) scan or magnetic resonance imaging (MRI) as part of their initial staging preoperatively. Informed consent was obtained from every subject.

### Surgery

The patients had lobectomy or ipsilateral pneumonectomy. Complete mediastinal lymph node dissection or systematic mediastinal lymph node sampling was performed during surgery. Complete resection was defined as resection of all macroscopic tumor and margins free of tumor at microscopic analysis.

### Chemotherapy

Adjuvant chemotherapy was routinely administered except for patients with asthenia or refusal to chemotherapy and given at the discretion of the treating physicians, with a cisplatin or paclitaxel-based regimen and a median of 4 cycles.

### Radiotherapy

Postoperative radiotherapy (PORT) was administered at the discretion of the attending radiation oncologist and the suggestion of the referring surgeon. Three-dimensional conformal radiotherapy (3DCRT) and conventional 2-dimensional radiotherapy (2DRT) were administered with a linear accelerator using 6–8 MV x-ray at 2 Gy per fraction, 5 days per week, to a total dose of 60 Gy.

### Follow-Up

Patients were followed up every 3 months for the first year, then twice a year for the following 2 years, and yearly thereafter. All patients were evaluated with a physical examination, complete blood count, serum biochemistry, thoracic CT scans, abdomen B-ultrasound examination, and other necessary examinations based on the patient’s symptoms. Follow-up brain MRI was performed upon development of suspicious symptoms or as part of restaging at the time of disease recurrence or yearly. Sites of disease progression or relapse were determined either radiologically or histologically. Both the initial and the subsequent sites of recurrence were documented.

### Data analysis

A failure event for overall survival (OS) was defined as death of any cause. A failure event for disease-free survival (DFS) was defined as the earliest event of death of any cause, locoregional recurrence, distant recurrence, or second primary tumor. Locoregional relapse was defined as progression or recurrence at primary lung sites, or within the hilar, mediastinal, or supraclavicular lymph node regions. A distant relapse was defined as a recurrence in the other regions outside the local area such as bone, liver, adrenal glands, contralateral lung, and brain. Time to event was measured from the date of surgery to the date of failure or to the date of last follow-up if no failure occurred. The cumulative incidence of BM and survival were estimated by Kaplan–Meier method. Log-rank test was used to compare the difference between groups. The Cox regression was used to assess the strength of association between time to development of BM and clinical and pathological risk factors. Variables included gender, age, smoking history, site of primary tumor, histology, tumor size, tumor stage, histologic grade, adjuvant chemotherapy, PORT, number of metastatic lymph nodes, ratio of metastatic to examined nodes or lymph node ratio (LNR), number of metastatic mediastinal lymph nodes, number of metastatic mediastinal lymph nodes station, and ratio of metastatic mediastinal lymph nodes station. The correlation between each of the variables was determined by the chi-square test. When two variables were significantly correlated, the variable more significantly linked to the risk of BM was included in the multivariate analysis. Multivariate Cox regression analysis assessed risk factors for development of BM. In the multivariate Cox regression model, all variables with P values < .10 in the univariate analysis were included. All statistical tests were two-tailed, and P < .05 was considered significant. SPSS 18.0 was used for all statistical analyses.

## Results

### Patient characteristics

Among the 221 patients included in the study, 4 were excluded due to incomplete data of relapse pattern. Therefore, 217 patients were analyzed. Median follow-up time for the 55 surviving patients was 71.3 months (range, 58.7–103.5 months). The patient and treatment characteristics are shown in Table 1. The median age was 60 years (range, 27–79 years). Adenocarcinoma (51.6 %) was the predominant pathological type, followed by squamous cell carcinoma (40.1 %). Of all patients, 159 (73.3 %) received adjuvant chemotherapy, and 93 (42.9 %) received PORT.

**Table 1:** Patient and treatment characteristics

**Characteristic**	**No.**	**%**
Gender		
Male	156	71.9
Female	61	28.1
Age		
≤60	114	52.5
>60	103	47.5
Weight loss		
≤5 %	208	95.9
>5 %	9	4.1
Smoking History		
Negative	98	45.2
Positive	119	54.8
Preoperative KPS score		
70	8	3.7
≥80	209	96.3
		
Preoperative hemoglobin		
<120 g/l	18	8.3
≥120 g/l	199	91.7
Preoperative clinical stage N2		
No	88	40.6
Yes	129	59.4
Histology		
Squamous	87	40.1
Adenocarcinoma	112	51.6
Adenosquamous	12	5.5
Large cell	6	2.8
Tumor stage		
T1	17	7.8
T2	163	75.1
T3	37	17.1
Laterality		
		
Left	96	44.2
Right	121	55.8
Location		
Upper/middle lobe	135	62.2
Lower lobe	82	37.8
Type of surgery		
Lobectomy	196	90.3
Pneumonectomy	21	9.7
Histologic grade		
Well/moderate	144	66.4
Poor	64	29.5
NS	9	4.1
Adjuvant chemotherapy		
No	58	26.7
Yes	159	73.3
Postoperative radiotherapy		
No	124	57.1
Yes	93	42.9
No. of dissected lymphonodes		
1-20	103	47.5
20-60	114	52.5
No. of metastatic lymphonodes		
≤5	112	51.6
>5	105	48.4
LNR		
≥ 30 %	110	50.7
< 30 %	107	49.3
No. of metastatic mediastinal lymphonodes		
< 3	107	49.3
≥ 3	110	50.7
No. of metastatic mediastinal lymphonodes station		
1	136	62.7
2-4	81	37.3
Ratio of metastatic mediastinal lymphonodes station		
≤50 %	108	49.8
>50 %	108	49.8
NS	1	0.5

KPS indicates Karnofsky performance status; LNR, lymph node ratio; NS, not stated.

### Survival and recurrence pattern

**Figure 1:**
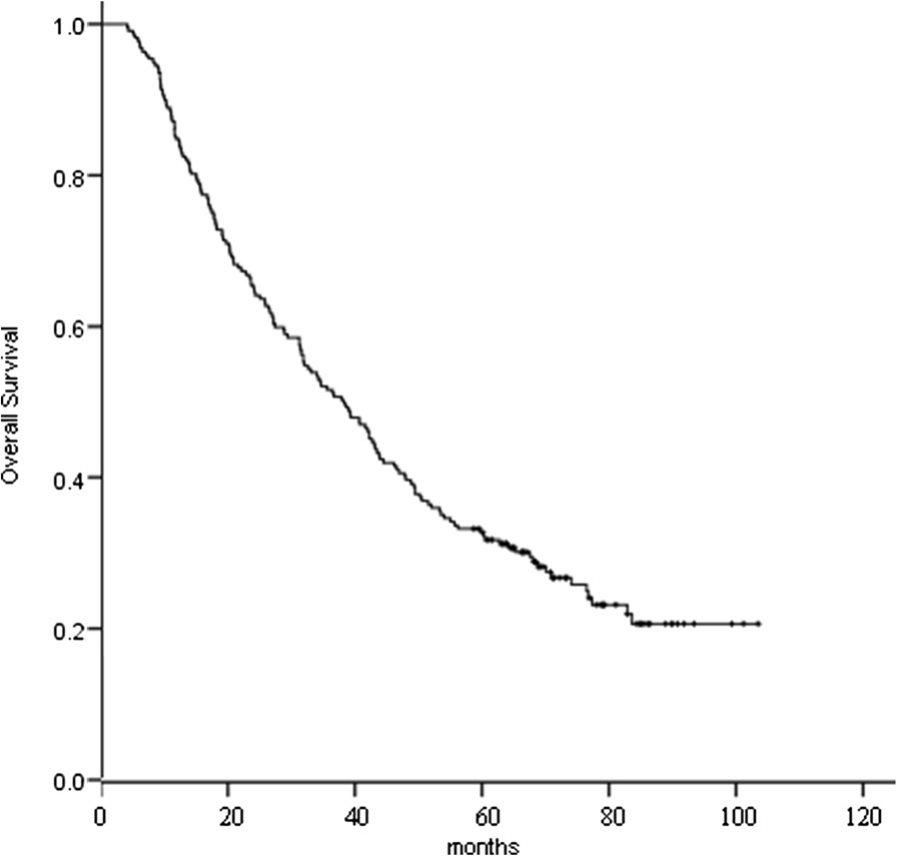
Overall survival for the 217 patients.

**Figure 2:**
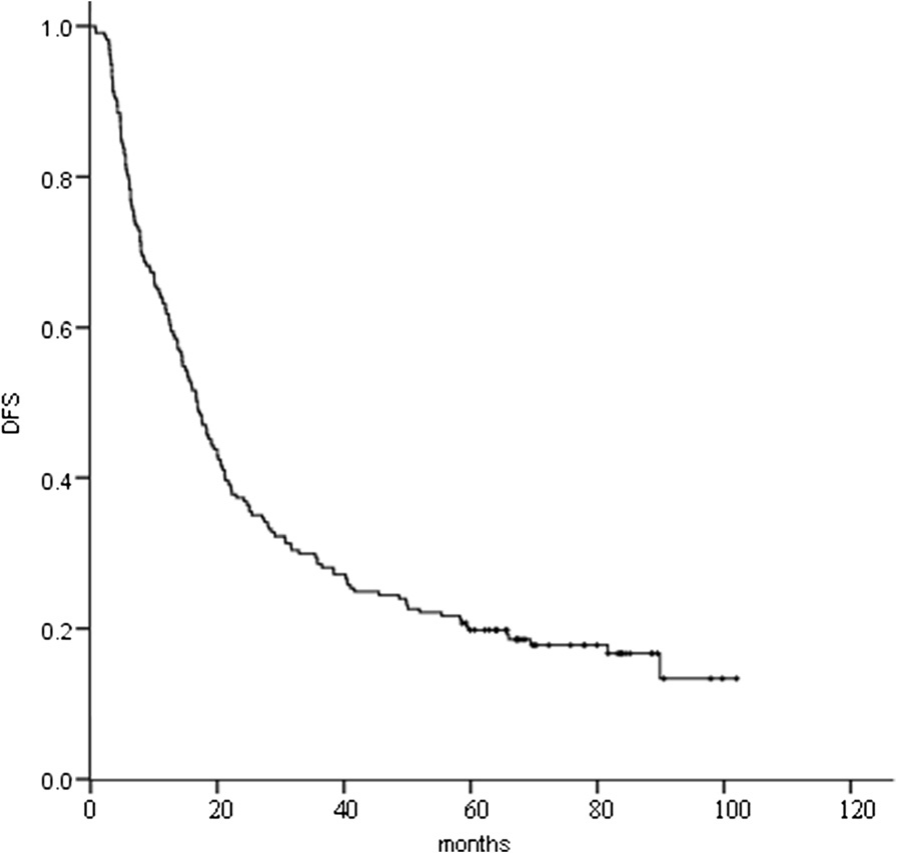
Disease-free survival (DFS) for the 217 patients.

Median OS for this patient population was 38.3 months. The 1-, 2-, 3-, and 5-year OS was 84.8 %, 65.4 %, 51.6 %, and 32.7 %, respectively (Figure 1), and the corresponding DFS was 61.8 %, 37.3 %, 28.6 %, and 19.8 %, respectively (Figure 2). The 5-year OS for patients developing and not developing BM was 15.1 % and 38.4 %, respectively (P = 0.002).

**Figure 3:**
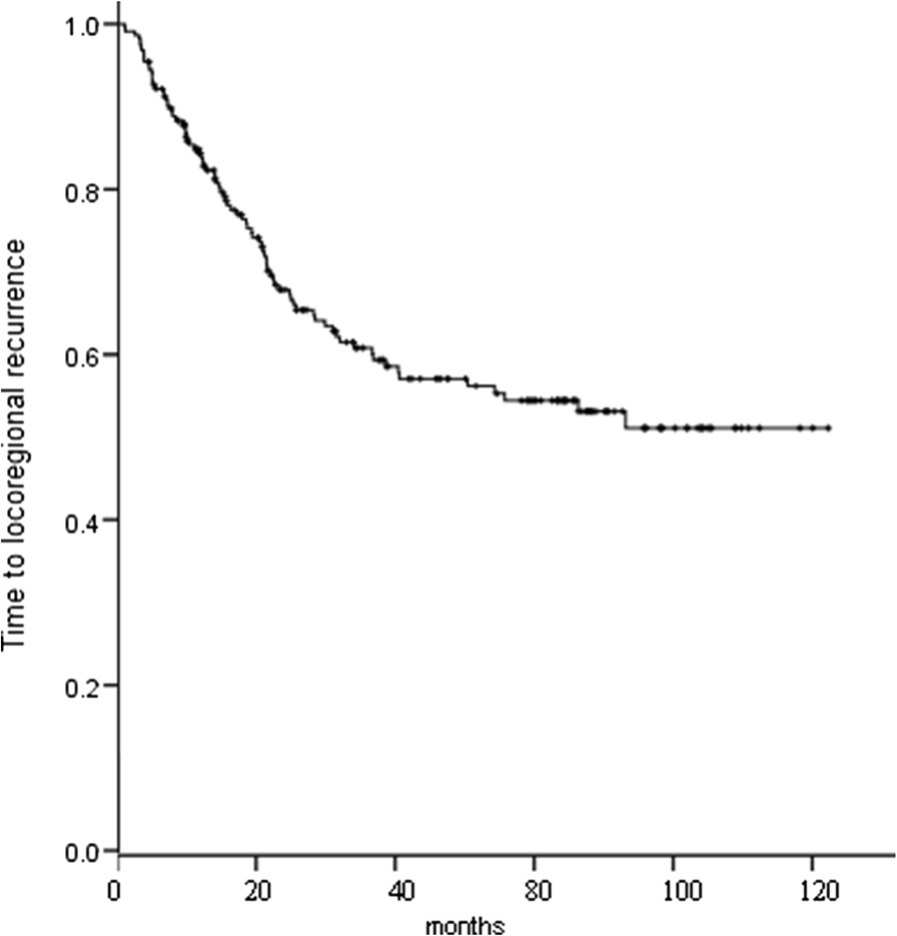
Time to locoregional recurrence for the 217 patients.

**Figure 4:**
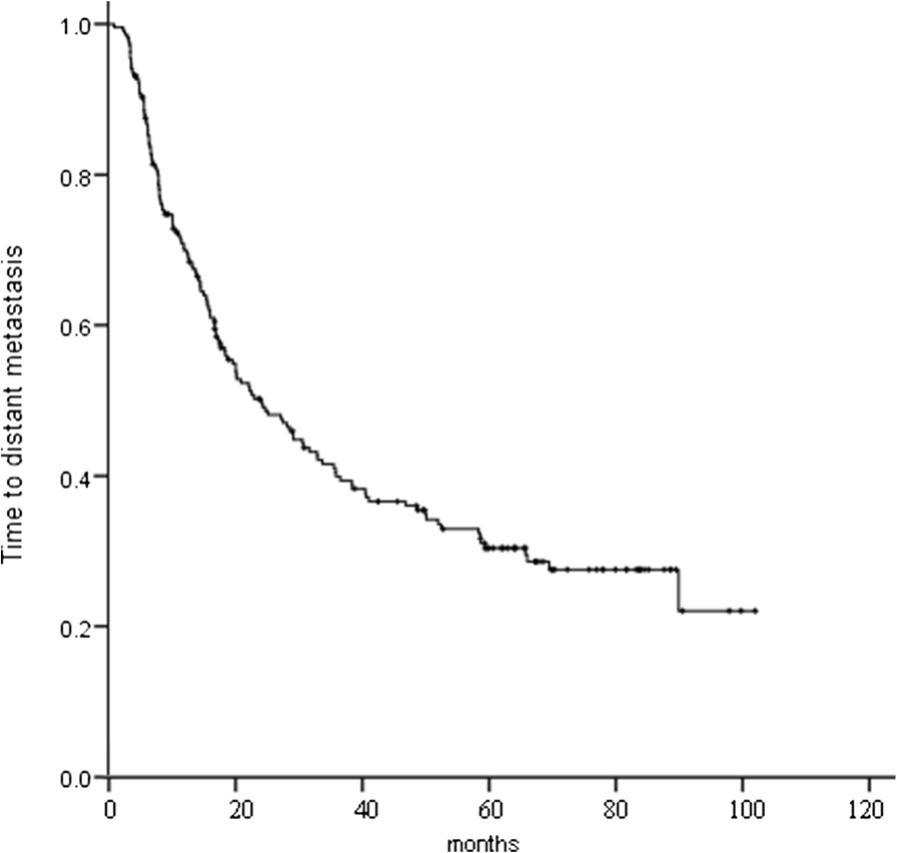
Time to distant metastasis for the 217 patients.

Up to the last follow-up, of all the 217 patients, local and/or distant recurrence was identified in 168 (77.4 %); first recurrence was local only (19.4 %), local and distant (12.4 %), and distant only (45.6 %); overall recurrence was local only (12.0 %), local and distant (26.7 %), and distant only (38.7 %). Of the 142 patients who had distant recurrences, 53 (37.3 %) developed BM. Figure 3 shows time to locoregional recurrence, and Figure 4 time to distant metastasis.

**Figure 5:**
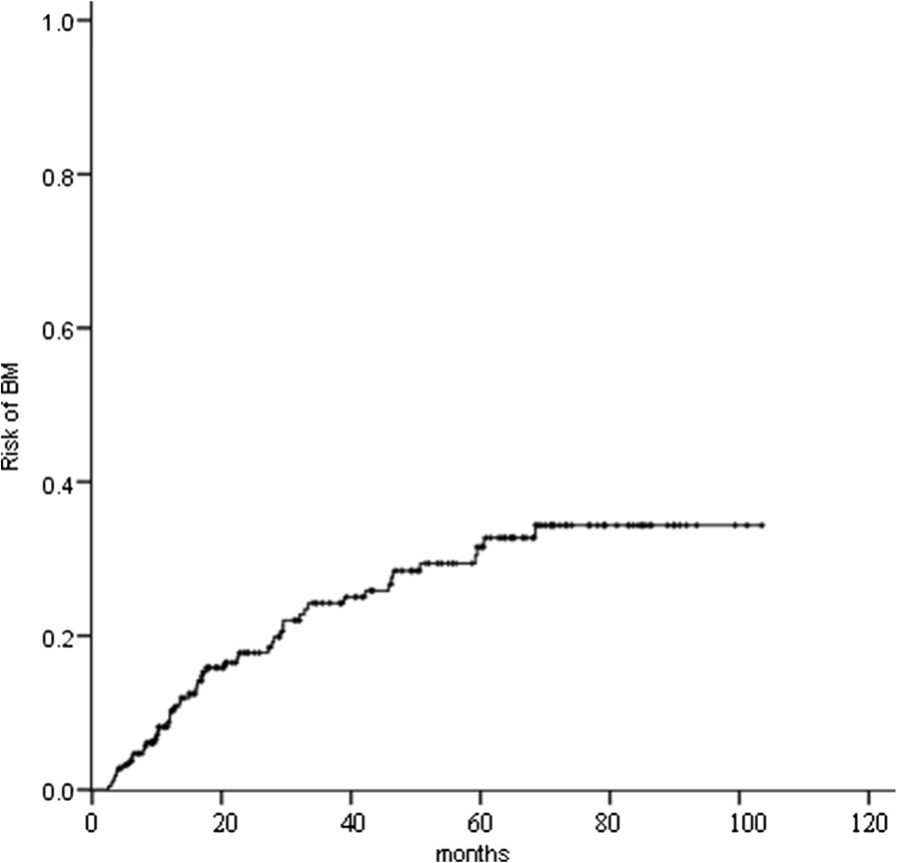
The actuarial risk of developing brain metastases (BM) for the 217 patients.

Of the 217 patients, 53 (24.4 %) developed BM at some point during their clinical course, 32 (14.7 %) recurred in the brain as their first site of failure, and 15 (6.9 %) recurred in the brain as their exclusive site of failure. The 1-, 3-, and 5-year actuarial risk of developing BM was 9.2 %, 24.2 %, and 31.5 %, respectively (Figure 5). Median time from surgery to onset of BM was 16 months (range, 2.5–68.5 months).

### Risk factors

**Figure 6:**
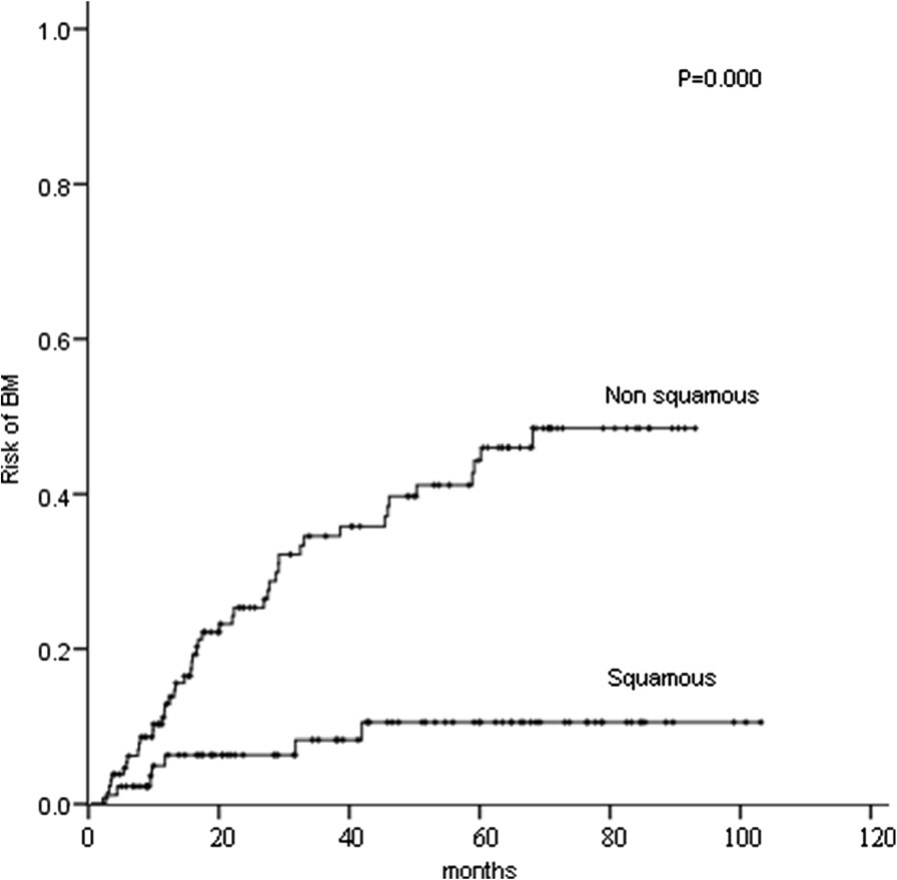
Comparison of the actuarial risk of developing brain metastases (BM) between squamous cell cancer and non squamous cell cancer.

**Figure 7:**
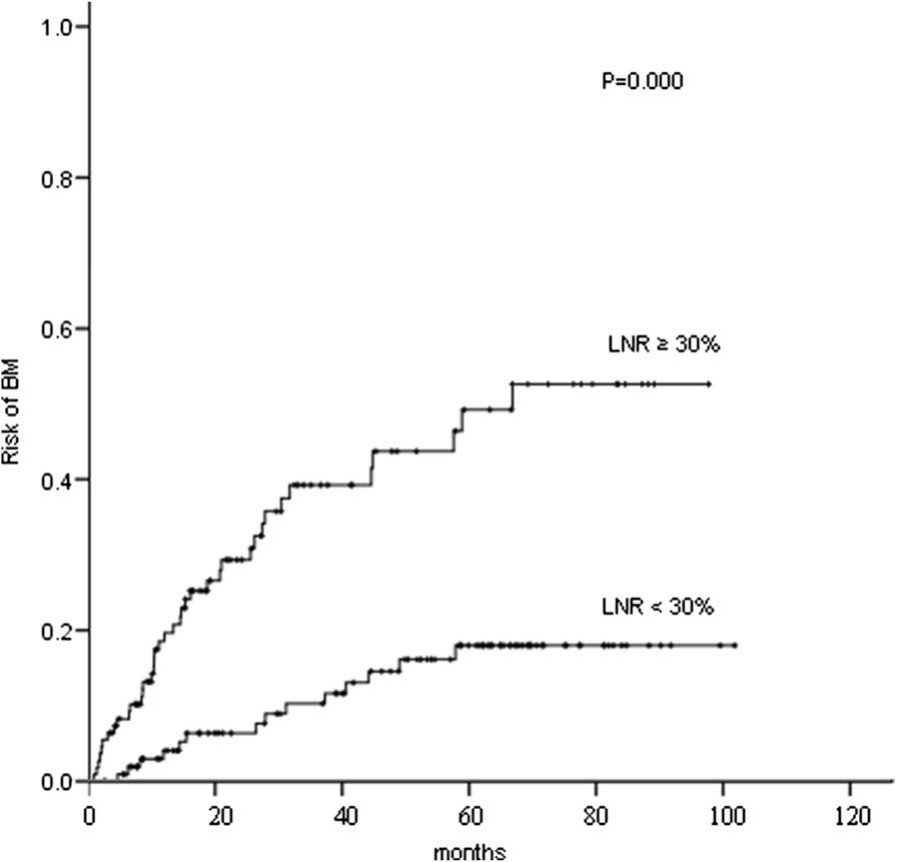
Comparison of the actuarial risk of developing brain metastases (BM) between lymph node ratio (LNR) ≥ 30 % and LNR < 30 %.

Several clinical and pathological factors were found to be associated with the development of BM on both univariate and multivariate analyses (Table 2). On univariate analysis, female (P = 0.012), age ≤ 60 years (P = 0.035), negative smoking history (P = 0.038), non-squamous cell cancer histology (P = 0.000) (Figure 6), number of metastatic lymph nodes > 5 (P = 0.000), LNR ≥ 30 % (P = 0.000) (Figure 7), number of metastatic mediastinal lymph nodes ≥ 3 (P = 0.001), number of metastatic mediastinal lymph nodes station of 2–4 (P = 0.000), and ratio of metastatic mediastinal lymph nodes station > 50 % (P = 0.000) were associated with an increased risk of developing BM. By chi-square test, the number of metastatic lymph nodes, number of metastatic mediastinal lymph nodes, number of metastatic mediastinal lymph nodes station, and ratio of metastatic mediastinal lymph nodes station were significantly correlated with LNR (P < 0.01). Therefore, only LNR, the variable most significantly linked to the risk of BM, was included in the multivariate analysis. On multivariate analysis, non-squamous cell cancer histology (RR: 4.13, 95 % CI: 1.86–9.19; P = 0.001) and LNR ≥ 30 % (RR: 3.33, 95 % CI: 1.79–6.18; P = 0.000) were associated with an increased risk of developing BM. Identically, on multivariate analysis, non-squamous cell cancer histology (RR: 4.34, 95 % CI: 1.51–12.44; P = 0.006) and LNR ≥ 30 % (RR: 2.35, 95 % CI: 1.10–5.02; P = 0.027) were associated with an increased risk of BM as the first relapse.

**Table 2:** Factors associated with the development of brain metastases

		**Univariate analysis**			**Multivariate analysis**	
		Incidence of BM (%)				
Factors	1-yr	3-yr	5-yr	P	P	RR (95 % CI)
Gender						
Male	7.6	20.5	26.9	0.012		
Female	13.3	33.4	43.0			
Age						
≤60 yrs	14.1	28.8	37.9	0.035		
>60 yrs	3.9	19.3	24.9			
Smoking History						
Negative	11.7	28.6	37.9	0.038		
Positive	7.2	21.0	26.8			
Histology						
Non- squamous cell cancer	12.0	34.6	44.3	0.000	0.001	4.13(1.86-9.19)
Squamous cell cancer	5.0	8.3	11.4			
Tumor stage						
T1	5.9	20.1	36.1	0.596		
T2	8.3	22.6	30.1			
T3	14.4	36.9	36.9			
Laterality						
Left	9.8	26.8	33.9	0.456		
Right	8.7	22.2	30.3			
Location						
Upper/middle lobe	7.8	22.3	31.7	0.798		
Lower lobe	11.5	27.2	31.6			
Histologic grade						
Well/moderate	8.8	21.7	31.1	0.601		
Poor	11.4	29.5	33.0			
Adjuvant chemotherapy						
No	11.0	21.2	24.7	0.511		
Yes	8.5	25.1	33.8			
Postoperative radiotherapy						
No	9.5	25.3	34.4	0.344		
Yes	8.7	23.0	29.0			
No. of metastatic lymphonodes						
≤5	4.6	13.3	20.9	0.000		
>5	14.5	38.6	46.3			
LNR						
≥ 30 %	15.3	39.2	46.4	0.000	0.000	3.33(1.79-6.18)
< 30 %	2.9	10.3	18.4			
No. of metastatic mediastinal lymphonodes						
< 3	4.9	13.8	21.0	0.001		
≥ 3	13.6	35.2	43.0			
No. of metastatic mediastinal lymphonodes station						
1	6.0	14.8	21.8	0.000		
2-4	15.1	42.7	51.6			
Ratio of metastatic mediastinal lymphonodes station						
≤50 %	3.8	13.8	19.5	0.000		
>50 %	15.0	35.8	44.9			

BM indicates brain metastases; RR, relative risk; LNR, lymph node ratio.

**Figure 8:**
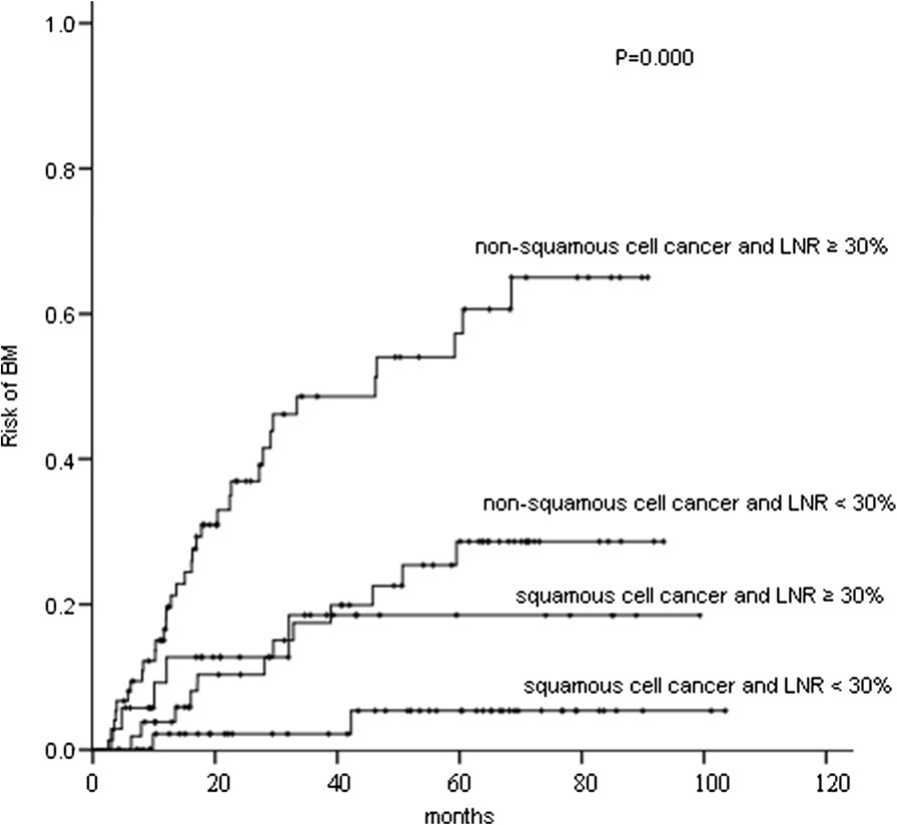
Comparison of the actuarial risk of developing brain metastases (BM) among patients with two, one, and none of the two risk factors, non-squamous cell cancer histology and lymph node ratio (LNR) ≥ 30 %.

In patients with non-squamous cell cancer and LNR ≥ 30 % (n = 75), the 1-, 3-, and 5-year actuarial risk of developing BM was 18.1 %, 48.6 %, and 57.3 %, respectively. In patients with non-squamous cell cancer and LNR < 30 % (n = 55), the 1-, 3-, and 5-year actuarial risk of developing BM was 3.8 %, 17.5 %, and 28.7 %, respectively. In patients with squamous cell cancer and LNR ≥ 30 % (n = 35), the 1-, 3-, and 5-year actuarial risk of developing BM was 9.2 %, 18.5 %, and 18.5 %, respectively. In patients with squamous cell cancer and LNR < 30 % (n = 52), the 1-, 3-, and 5-year actuarial risk of developing BM was considerably lower (2.2 %, 2.2 %, and 5.3 %, respectively). The differences among the groups with both, one and none of the two risk factors were statistically significant (P = 0.000) (Figure 8).

## Discussion

With advances in surgical and radiation techniques and chemotherapy, combined-modality therapy for LA-NSCLC has led to improvements in locoregional and extracranial distant control, therefore in OS. Nevertheless, the risk of BM remains. In our study, of the 217 patients, 53 (24.4 %) developed BM at some point during their clinical course, and 32 (14.7 %) recurred in the brain as their first site of failure, and 15 (6.9 %) recurred in the brain as their exclusive site of failure. The 1-, 3-, and 5-year actuarial risk of developing BM were 9.2 %, 24.2 %, and 31.5 %, respectively. Our median time from surgery to onset of BM was 16 months (range, 2.5–68.5 months), longer than the reported 5.7–11.7 months [8-10,16,22,23]. In Stage IIIB NSCLC patients treated with PCI, lower BM and longer survival resulted from immediate concurrent chemoradiotherapy rather than induction chemotherapy-first regimens, which indicated the benefit of earlier PCI without delay because of induction protocols.[24] BM has a profound impact on morbidity and mortality.[9,11] Most patients who develop BM die of symptomatic intracranial tumor progression.[25,26] As extracranial control improves for LA-NSCLC, prophylaxis of BM becomes increasingly important.

Prior randomized [17-20] and nonrandomized [12,27-31] trials evaluating PCI for NSCLC have consistently reported a significant decrease and/or delay in BM, but no benefit in OS. The latter has been attributed to poor locoregional and extracranial control and/or small study size. RTOG-0214 is the only randomized, controlled trial to investigate PCI in LA-NSCLC in the modern era of combined-modality therapy. In RTOG 0214, patients with stage IIIA to IIIB NSCLC were eligible if they had stable disease or better after potentially curative therapy, defined as high-dose thoracic radiation therapy (RT; i.e., >30 Gy) or surgery.[20] The inclusion eligibility did not select the highest-risk population. Firstly, all kinds of NSCLC histology were included. Secondly, locoregional and extracranial distant relapse remains the major concern of the eligible stage IIIB and non-radically treated stage IIIA. Complete resection is a major curative therapy. Yet, only approximately 35 % of all patients underwent surgery, and the proportion of complete resection was not provided. Besides, it is debatable to define RT > 30 Gy as a potentially curative therapy. The risk of BM in the patient population of RTOG 0214 was not high enough to obtain a survival benefit from PCI. It is likely that locoregional and extracranial control was so poor that BM lacked the opportunity to manifest themselves. Future studies assessing PCI for NSCLC should be aimed at the highest-risk patients with satisfactory locoregional and extracranial control. Patients with stage IIIA (N2) made the radar. Moreover, radical resection is the most important curative treatment. In addition, primary surgery could provide accurate pathological stage. Hence, we targeted patients with completely resected pIIIA-N2 NSCLC.

Previous reports on risk factors of BM in NSCLC are inconsistent. The reported risk factors of BM in NSCLC include histology, [12,14-16,21,24] extent of disease,[10,13,22] adjuvant/neoadjuvant therapy, [12-15,22] younger age, [8,10,23] incomplete resection,[13]and carcinoembryonic antigen serum level[32]. In our series, on multivariate analysis, non-squamous cell cancer histology and LNR ≥ 30 % were associated with an increased risk of developing BM and BM as the first relapse. Non-squamous cell cancer as risk factor of BM and BM as the first relapse is consistent with many,[11,13-15,20,23,32-36] but not all,[7,8,10,12,19,22,31,37-39] previous studies.

It is generally understood that patients with more severe nodal metastases, including stage, number, size, and region of metastatic lymph nodes, are at a higher risk for BM. Several studies reported increased BM risk correlated with increasing lymphonode stage.[1,23,30,34,35] Besides, Ceresoli et al. observed that clinical bulky (≥2 cm) mediastinal lymph nodes was of borderline significance in predicting an increased risk of BM in IIB–IIIB NSCLC after multimodality treatment.[10] Wang et al. reported that a greater number of mediastinal lymph nodes and nodal regions with metastases predicted a higher risk of BM for stage III-N2 NSCLC.[13] Mamon et al. reported that the incidence of BM increased independently by the presence of residual nodal disease in 177 surgically treated IIIA (N2) NSCLC with/without neoadjuvant/adjuvant therapy.[11] In our study, on multivariate analysis, LNR ≥ 30 % was associated with an increased risk of BM and BM as the first relapse. We are the first to introduce LNR into the assessment of risk of BM in NSCLC, and to find its predictive value. Recently, Matsuguma et al. reported that the LNR followed by the number of metastatic nodes may be more effective prognostic indicators than the current nodal classification based on the location of metastatic nodes, and should be considered in the future nodal classification of lung cancer.[40] Another very recent study using Surveillance, Epidemiology, and End Results (SEER) database confirmed that the LNR is an independent prognostic factor in patients with N1 NSCLC and suggested that LNR may be used to identify patients who are at greater risk of cancer recurrence.[41] We could not compare the predictive efficacy of LNR and the current nodal staging here, as our study focused on a homogenous population of completely resected pIIIA-N2 NSCLC. Nonetheless, LNR might be a strong addition to the current nodal staging in predicting the risk of BM.

Tumor status is a major prognostic factor in NSCLC. Larger tumor size is associated with an increased risk of BM.[39] Bajard et al. demonstrated that in a cohort of 305 stage I–IIIB patients, T4 was related with a higher risk of BM, compared with T1–3.[34] However, many other investigators did not find that T status was significantly related with the risk of BM.[10,12,14,15] We did not find such a relation, either. The studies that did not find the relation have a distinguishing characteristic in common: they almost exclusively included LA-NSCLC cases, which may explain the inconsistent results.

Multiple reports have demonstrated that systemic therapy increases the BM risk and prolongs OS. Cox et al.[15] and Wang et al.[13] indicated that adjuvant chemotherapy increases BM incidence in LA-NSCLC. In addition, several investigators [9,12,14,22] found that an increased incidence of BM in patients treated with surgery is associated with neoadjuvant chemotherapy. Chemotherapy was not a statistically significant factor in our series. One of the reasons may be that the majority of our patients had undergone chemotherapy.

In our study, in patients with non-squamous cell cancer and LNR ≥ 30 % (n = 75), the 1-, 3-, and 5-year actuarial risk of developing BM was 18.1 %, 48.6 %, and 57.3 %, respectively. Such a subset had sufficiently common BM rates to justify future trials on PCI. We are planning a randomized, controlled trial to investigate PCI in the highest-risk subset we identified. In that future study, we may consider including surgically staged IIIA (N2) non-squamous cell cancer with residual nodal involvement after neoadjuvant therapy based on the study by Mamon et al. [11].

Previous studies on risk factors of BM in NSCLC often include heterogeneous populations with respect to pre/postoperative stage and treatment, whereas we targeted a homogeneous population. In addition, compared with the previous studies, our data have more detailed information on the extent of nodal metastases and other clinical characteristics. Furthermore, to our knowledge, our report is the largest retrospective study focusing on risk factors of BM in completely resected pIIIA–N2 patients from a single institution, especially within a relatively recent and short time period (2003–2005). On the other hand, like all other retrospective analyses, our study has its limitations. The study may have selection bias and the results should be interpreted cautiously. Nevertheless, the high follow-up rate of 217 of 221 consecutively treated patients assures that the patients studied are highly representative of the patients with completely resected pIIIA–N2 NSCLC at our institution. Additionally, long follow-up and accurate pathological stage after primary surgery both could help draw a relatively convincing conclusion.

## Conclusions

In NSCLC, patients with completely resected pIIIA-N2 non-squamous cell cancer and LNR ≥ 30 % are at the highest risk for BM, and are most likely to benefit from PCI. Further trials on PCI should focus on high-risk subset.

## Competing interests

The authors declare no competing interests.

## Authors' contributions

XD drafted the manuscript. XD, HD, ZH, and JL participated in the coordination of the study, and helped to analyze the data. LW conceived of the study, participated in its design, and helped to analyze the data. All authors made substantial contributions to acquisition of data, and read and approved the final manuscript.

An abstract about this work has been selected for Poster Viewing presentation during the 2011 ASTRO Annual Meeting.
